# 我国胸外科发展现状分析

**DOI:** 10.3779/j.issn.1009-3419.2014.07.02

**Published:** 2014-07-20

**Authors:** 逊 张

**Affiliations:** 300051 天津，天津市胸科医院 Tianjin Chest Hospital, Tianjin 300051, China

近年来，我国的胸外科事业取得了长足的进步，在某些领域达到世界领先水平。发展不平衡仍然是我国胸外科专业面临的问题。如何扬长避短，使我们的优势部分进一步加强，让短板的部分尽快发展，成为我们当前的一项重要工作。这首先需要对我国的胸外科发展现状有一个比较清楚的分析。

## 1 我国肺癌外科治疗现状分析

我国肺癌的发病率居高不下，肺癌的手术例数逐年增加。肺癌的微创外科治疗病例明显增加。根据国内几个大的医疗中心的资料分析，我国手术治疗的肺鳞癌病例逐年减少、肺腺癌的病例逐年增加。上海市肺科医院手术切除肺癌组织学类型的变化趋势显示：至2011年，手术治疗肺癌患者中鳞癌所占比例＜35%、腺癌比例增加到50%以上。随着我国健康普查的开展尤其是得益于低剂量螺旋计算机断层扫描（computed tomography, CT）的使用，手术治疗的肺癌患者中早期肺癌病例逐渐增加。上海市中山医院数据显示手术治疗的小结节病变比例逐年增加，至2010年，达到22.8%。该院手术治疗的Ⅰ期肺癌比例超过50%。上海市肺科医院资料显示近10年来手术治疗的Ⅰ期肺癌比例逐年增加，至2011年，手术治疗的Ⅰ期肺癌比例超过50%，而Ⅲ期肺癌的比例降低到30%以下。肺癌手术的并发症发生率和手术死亡率明显降低，但是肺癌微创手术的远期生存率从总体上讲还没有明显提高。北京大学肿瘤医院胸外科（陈克能）单纯手术组pTNM生存分析（*n*=444）：Ⅰ期5年生存率为81.9%、Ⅱ期为52.1%、Ⅲ期为64.6%。上海市中山医院（王群）肺叶切除术远期疗效总体5年生存率59.7%。今后应积极开展以胸外科为主的早期肺癌的临床研究，进一步提高肺癌手术的疗效。

## 2 肺切除微创外科治疗现状分析

目前开展的胸腔镜肺切除术的几种方法有四孔法、三孔法、单操作孔法和单孔法，其中以三孔法为主流。美国国立综合癌症网络（National Comprehensive Cancer Network, NCCN）非小细胞肺癌治疗指南（2014版）指出，大量证据表明，电视胸腔镜手术（video-assisted thoracoscopic surgery, VATS）肺叶切除术可以改善早期结果（减少疼痛、减少住院时间、减少并发症、更快地康复）。因此，在没有反指证的情况下、积极推荐使用VATS肺叶切除术。近几年来，我国胸腔镜肺切除术的比例逐年增加。上海市胸科医院2013年行肺切除手术4, 231例，其中胸腔镜肺切除术2, 466例，占58%。上海市肺科医院2013年肺切除手术3, 060例，其中VATS微创手术2, 040例占66.7%。上海中山医院胸外科胸腔镜在肺癌切除中的比例达到了87.6%。除了胸腔镜手术比例不断增加外，近几年，我国在胸腔镜肺切除术的提高方面也取得了多项成绩：刘伦旭（华西医院）完成国际上首创全腔镜支气管、肺动脉双袖式切除成形术（迄今已成功完成4例），并在国际上首创胸腔镜出血有效止血技术-“腔镜吸引侧压止血技术”，还首创了处理腔镜胸膜腔粘连的“隧道”法。付向宁（武汉同济医院）创新VATS肺段边界标记新方法-“美兰标记法”：切断肺段支气管后，注入稀释的美兰，肺段边界自然显现，由于注入的美兰不易与其它肺段交通，此方法较传统膨肺法能够更加准确切除肺段。

何建行（广州医学院第一医院）成功开展了非插管硬膜外麻醉胸部手术，术式包括了肺叶切除术、肺楔形切除术、肺大疱切除术、肺减容术、纵隔肿瘤切除术、重症肌无力手术、手汗症微创手术、漏斗胸矫形手术等。非插管硬膜外胸部手术好处包括：①避免了插管对气管支气管的机械损伤；②避免了肌松剂对全身肌肉的抑制及随后液体容量扩充引致心肺负荷增加；③避免了呼吸机人工通气对双肺所产生的机械损伤。

目前我国肺切除微创技术发展存在的问题：①发展不平衡，各医疗机构之间微创技术水平相差甚远；②缺乏国内统一胸腔镜各类手术的技术标准及规范；③多数县级医院腔镜设备均较为陈旧，不适合开展腔镜肺叶切除手术；④年轻医师缺乏训练机会；⑤目前手术器械主要依靠国外进口，自主研发的相关器械较少且技术含量不足，造成手术费用相对较高；⑥有效并规范化的腔镜技术培训亟待解决，目前主要依赖企业支持；⑦医疗保险的限制也制约了胸腔镜技术的普及和提高。

## 3 我国气管外科现状分析

气管外科是体现普胸外科手术技术水平的亚学科。气管外科的特点是手术设计及麻醉操作困难、术中解剖复杂需精细操作、术后并发症较高。气管外科目前已获批的国家自然基金课题数25项，共计1, 009.6万元，其中近3年相关课题为13项。气管外科可能成为我国胸外科在短期内出现国际重大突破的领域。姜格宁（上海市肺科医院）近年来开展了多项气管外科创新技术：新型隆突重建手术（全球首创/改进）、人工气管置换术、自体支气管瓣修补气管成形术（国内首例）、自体肌皮瓣修补气管缺损、自体肋软骨修补颈部气管长段狭窄的术后缺损、自体带蒂胸大肌肌皮瓣移植和气管节段切除治疗长节段气管肿瘤（全球首例）。

李简（北京大学第一医院）国际首创了分期异体气管移植术，迄今已经成功完成了3例。其中2例为食管癌侵及气管、1例为原发性气管肿瘤长度超过6 cm的患者。手术分为二期进行。第一期在患者的大网膜内植入了异体气管，术后给予免疫抑制剂。21天后确认气管上的血管再生，说明气管存活，再进行第二阶段手术：重新打开腹部，取出已植入的带有血管网膜蒂的移植气管，连同大网膜移至胸腔，然后行气管肿瘤根治性切除，并用移植的气管对损伤的气道进行重建。3例患者术后均康复出院。术后6个月气管镜检查提示3例移植气管均被覆正常气管粘膜上皮。对于原发气管肿瘤长度较大的患者，本术式是一种安全可行的方法。对于食管癌侵及气管的患者，这种异体气管移植+结肠代食管手术是一种可选择的手术方式。付向宁（武汉同济医院）首创了左主支气管根部延长、隆突重建治疗累及隆突右侧壁的中央型肺癌术式。并首创了胸腔镜-开放杂交式同期双侧手术行全隆突切除+袖式左全肺切除术。该术式适于左主支气管癌侵及隆突患者，先右侧开胸离断左主支气管，行隆突切除+右主支气管-气管吻合，这样避免了在左胸行隆突切除重建。右胸术后，再行胸腔镜左全肺切除+淋巴结清扫术。李小飞（第四军医大学唐都医院）国际首创了“双瓣式”食管-气管瘘修补术，用简单的方法解决了复杂的气管瘘的修补问题。迄今已完成62例手术，成功率100%。我国气管外科目前存在的问题：国内开展气管外科手术的医疗机构极少，且水平普遍不高；气管外科基础研究亟待提高；组织工程气管距运用于临床依然相距甚远。

## 4 我国肺移植的现状分析

肺移植是治疗终末期肺实质及肺血管病变唯一有效的方法。由于肺脏是相对容易损伤的器官，是唯一直接与外界接触的生命器官，以及肺移植受者的复杂性与术前基本情况相对较差等原因，肺移植技术在各主要器官移植中成功最晚。目前全球肺移植数量每年已经超过3, 500例。1978年辛育龄教授在我国开展了第1例肺移植手术，1978年-2013年全国肺移植统计共计完成438例。无锡市人民医院（陈静瑜）完成的肺移植数量居国内首位，其2013年达到67例（双肺移植52例、单肺移植15例）。上海市肺科医院（姜格宁）开展的肺移植数量虽在国内第二，但是国内唯一能够开展各种肺移植术式的单位，包括国内首例活体肺移植、亚洲首例肺移植术后再移植、亚洲最大年龄患者（75岁）肺移植；手术死亡率9.7%，居国内领先水平；5年生存率达到60.2%，为国内最好水平。目前我国每年肝移植总数为1, 500例，肾移植3, 000例左右，而肺移植每年仅有50例-60例，仅利用了3%的供肺资源，即我国目前有大量的肺资源被浪费。目前我国已经建立了“中国肺移植注册系统”，进行肺脏分配与共享，从而能够更合理、有效地利用肺资源。

我国肺移植手术目前存在的问题：①国内肺移植中心水平存在明显不均衡，全国有27家获批的肺移植机构，仅5家医院在规模开展肺移植手术，多数移植中心盲目追求手术数量，无法确保患者的长期存活及生存质量；②肺移植的基础研究和转化研究需要进一步加强。建议进行优势资源力量整合，划片区建立大型移植中心；③器官捐赠流程及供体分配制度有待进一步细化、完善；④由于大多数供体存在不同程度的肺部感染和损伤，需引进国外最先进的体外处理技术（国外仅几家医院掌握此技术）。加快国内这项技术的开发和引进，进一步提高我国肺移植的成功率和远期生存率。

## 5 达芬奇机器人在我国胸外科应用现状分析

1997年达芬奇机器人研制成功，2000年获得美国食品药品监督管理局（Food and Drug Administration, FDA）批准用于临床。达芬奇外科手术辅助系统代表了目前微创外科尖端技术。达芬奇机器人有以下几点优势：①突破人手局限的可转腕器械，有6个关节、7个方向，可完全模仿人手动作，360o活动。在狭窄解剖区域中比人手更灵活；②直观实时的动作控制，可滤除人手颤抖，充分利用开放手术经验，易于掌握，学习曲线短；③突破人眼局限的成像系统，三维立体腔镜放大10倍-15倍的高清晰立体图像。至2013年12月全球共有2, 966台机器人用于临床。其中美洲2, 083台、欧洲476台、亚洲288台。目前我国内地共有18台机器人，分布在14家医院。至2013年12月，我国累计完成机器人手术6, 535例，其中胸外科累计完成607例。至2013年12月，国内共有10家医院开展了机器人普胸手术，手术数量前5位的医院分别为沈阳军区总医院（260例）、南京军区总医院（191例）、上海胸科医院（70例）、第二炮兵医院（49例）、301医院（11例）。2014年*J Thorac Cardiovasc Surg*杂志发表了Scott J. Swanson等的一篇文章：“机器人辅助胸腔镜肺叶切除或楔形切除术与传统电视胸腔镜手术的比较：来自多医院Premier数据库的结果”。这项研究代表了最新和最广的评价机器人手术和胸腔镜手术成本效益结果的分析。作者对305家医院Premier数据库（15, 502例）机器人与胸腔镜肺切除术进行了比较。研究结果显示：无论肺叶切除术或楔形切除术，较之传统电视胸腔镜手术，机器人手术有着更高的住院费用和更长的手术时间，而二者的并发症发生率没有差异。这使人们对于这项技术的成本效益提出质疑。使用机器人需要进一步考虑费用与效益之间的关系。对我国机器人手术的成本效益分析，由于开展的时间短，开展的医院也少，还无法得出有说服力的结果。目前在我国机器人的购置费用为2, 000万-2, 500万元人民币，机器人维护费用每年在100万元左右；目前国内医院机器人的开机费用为每次1.5万-2万元；机器人胸外科手术每次需用2把-3把专用手术器械，每把手术器械的价格为6.5万-7万元，每把器械可用10次，需要平摊在每次手术费用中。以上海市胸科医院肺叶切除术为例，机器人肺叶切除术的费用比胸腔镜肺叶切除术的费用平均多4万元左右。由于手术费用的昂贵，机器人胸外科手术目前在我国普及有许多困难。近两年，3D胸腔镜开始进入我国市场。与普通电子胸腔镜相比较，前者为仿真三维立体之外，可以将结构放大10倍；后者为二维平面，结构放大3倍-5倍。在使用手术器械和高质耗材上两者没有区别。对于初学者来说，3D胸腔镜的操作感觉更接近于开放式手术，因此，学习曲线短，更容易掌握。对于已经熟练掌握了普通电子胸腔镜的医生来说，原来已经适应了二维平面下的手术操作，如使用3D胸腔镜，反而需要一个再适应的过程。由于3D胸腔镜的购置费用需要400万元人民币左右，因此，患者的医疗费用要高于普通电子胸腔镜。3D胸腔镜今后的发展趋势还需要进一步在实践中观察。

## 6 我国食管外科现状分析

我国食管外科近几年的发展，主要集中在食管微创手术方面。微创食管癌外科术式主要有：①三切口微创手术：为目前的主流术式，迄今国内已经完成了数千例。其又分为“胸腔镜+开腹+颈部吻合术”和“胸腔镜+腹腔镜+颈部吻合术”两种术式；②微创Ivor-Lewis手术：目前为非主流术式，迄今国内完成不足500例，又分为“胸腔镜+腹腔镜”和“胸腔镜+开腹”两种术式。

与肺切除微创手术相比较，食管的微创手术仍然处于普及推广阶段。目前主要在少数大的医院开展。胸腔镜食管癌切除术普及困难的原因分析：①由于食管癌手术自身的复杂性，既要进行食管癌切除、淋巴结清扫，还要重建消化道，手术时间要明显长于胸腔镜肺叶切除术；②技术难度大，要求术者具有丰富的常规手术经验，同时掌握各类胸、腹腔镜操作，学习曲线较胸腔镜肺叶切除术相对更长；③食管癌手术后并发症发生率明显高于肺叶切除，因此，在目前医患关系紧张的环境下，开展新技术要冒更大的风险；④食管癌微创手术的高质耗材费用要明显高于肺叶切除术，高额的医疗费用也成为制约微创食管癌手术的一个重要因素。虽然有上述的种种困难，胸腔镜食管癌切除术在我国还是逐年增加的。上海市中山医院（王群、谭黎杰）至2013年12月已累计开展胸腔镜食管癌切除术700余例。其中2013年胸腔镜食管癌切除术占同期食管癌手术的40%左右。上海市胸科医院2013年胸腔镜食管癌切除术占同期食管癌切除术的13.4%。

胸腔镜胸内食管胃吻合器吻合（Orvil）仅在少数医院开展，迄今开展了100余例。Orvil食管-胃胸内吻合术开展困难的原因分析：①腔镜下胸内食管-胃吻合相对颈部开放性手术更复杂和困难；②由于与传统的吻合方式不同，胸外科医师的接受程度受影响；③受微创食管外科整体普及较差的影响，开展范围受限；④大多数地区，该器械未能进入招标目录。Orvi手术的应用前景分析：不会成为胸内胃食管吻合的唯一微创技术，但至少是一种技术方法之一。付向宁（武汉同济医院）应用普通吻合器行全腔镜下Ivor Lewis手术。先腹腔镜游离并制作管状胃，置于腹腔备用。再经右胸完全胸腔镜切除食管，普通吻合器胸内吻合（微创Ivor Lewis）。至今已完成200例，吻合口瘘发生率2%，均治愈。

传统上我国食管癌切除术以Sweet术式（左胸手术）为主，近些年来，Ivor Lewis术式（右胸+开腹）逐年增加。2014年王群等在*Ann Thoracic Surg*杂志发表文章，通过对上海中山医院食管癌的资料分析，显示左、右开胸食管癌切除术的远期生存率是相等的。提出左胸食管癌根治术的合理应用，认为中下段早期食管癌适宜左开胸。根据美国SEER数据库1998年-2003年742例0期、Ⅰ期食管癌资料分析，早期食管癌内镜下切除术（99例）和外科手术切除术（643例）的远期生存率没有明显差异。近年来，内镜下早期食管癌切除术也开展了起来，但目前主要是内镜医生进行，胸外科医生进行此项治疗的不多。上海市中山医院食管癌的内镜切除术是国内开展最多的单位，现在每年可达200余例。其远期疗效有待进一步观察。傅剑华（中山大学肿瘤防治中心）负责主持的“术前放化疗并手术与单纯手术治疗局部晚期食管癌的多中心（8家医院）随机对照研究”，目前已经取得了中期结果。采用长春瑞滨+顺铂和同期常规分割放疗（总剂量40 Gy），截止目前的研究结果显示，术前放化疗对局部晚期食管鳞癌具有明显的降期疗效、取得较高的pCR率、可提高肿瘤的完全性切除率。术前放化疗组与单一手术组相比较，3年生存率差异明显（72.5% *vs* 50.9%, *P*=0.021）。本项研究显示术前放化疗对MIE手术分离的影响，优势是使肿瘤缩小，易于分离；毛细血管闭塞，出血减少。同时，由于放化疗反应致纤维化形成，导致食管周围解剖间隙不清，增加气管膜部损伤机会，增加了手术难度。而选择合适的手术时间尤为重要，以放疗后4周-8周为宜。近年来，食管良性疾病的微创外科治疗也取得了明显的成绩。天津市胸科医院（张逊）采用杂交式手术（首先介入治疗给予带膜的主动脉支架，再开胸游离缝合食管-主动脉瘘口）成功抢救食管主动脉瘘患者。2012年12月，张逊等在国际上首创采用胸腔镜治疗巨大咽食管憩室获得成功。

## 7 我国纵隔镜与气管腔内超声（endobronchial ultrasonography, EBUS）应用分析

纵隔镜目前仍是纵隔占位诊断的最佳手段，对肺癌纵隔淋巴结分期，非癌性病变或EBUS无法确诊者仍依赖纵隔镜检查。EBUS通过支气管内超声引导纵隔淋巴结（肿块）针吸活检，评估纵隔淋巴结转移及纵隔不明性质包块的诊断。对肺癌纵隔分期的价值不亚于纵隔镜检查，且创伤小，可门诊检查，但对非癌性病变诊断率相对较低。上海市胸科医院纵隔镜、气管腔内超声近3年应用统计显示，2011年-2013年，纵隔镜检查例数逐年减少（97例-79例），而EBUS应用量逐年增加（439例-713例）。有学者认为EBUS有取代纵隔镜在肺癌分期中地位的趋势。电磁导航支气管镜可实现经纤支镜对肺内周边小结节准确活检，该项设备即将正式进入我国市场，此前，国内仅有1家医院在临床试用。

## 8 胸壁畸形微创外科治疗的现状分析

胸壁畸形主要包括漏斗胸、鸡胸。近10年来治疗这两种胸壁畸形的NUSS手术在我国迅速得到普及。1998年Nuss报道漏斗胸微创外科治疗，2002年国内开展了NUSS手术。目前全国有上百家医院开展了漏斗胸的NUSS手术治疗，手术例数超过了1万例。北京儿童医院成立了NUSS手术中国培训中心，2007年以来每年举办全国NUSS手术学习班，有力地促进了NUSS手术在我国的推广。曾骐（北京市儿童医院）改良了8种手术方法，手术数量为世界第一。至2014年3月，该院手术治疗漏斗胸2, 610例，有效率达到100%；手术治疗鸡胸220例，有效率达到99.34%。NUSS手术在我国开展存在的问题：随着手术应用范围扩大、手术者不断的增加，出现了致命的并发症，因此，手术方法有待规范。

## 9 手汗症的微创外科治疗现状

以涂远荣（福建医科大学附属第一医院）为组长，由25个协作单位组成的中国手汗症微创治疗协作组经过10余年的努力，目前我国关于手汗症的整体研究水平和微创外科治疗处于国际先进水平。该协作组进行的手汗症发病机制的基础研究、手汗症遗传家系及流行病学的调查研究和原发性手汗症（primary palmar hyperhidrosis, PPH）家系致病基因的定位克隆的研究取得了可喜的结果。手汗症的微创手术方法不断改进：麻醉由双腔插管、单腔插管到喉罩或面罩静吸复合全麻。切口由传统的2个-3个切口改进为经乳晕或腋窝单切孔。胸交感神经切断平面改进，将传统的R2-4切断术改进为保留R2，仅作R3切断术，使术后代偿性多汗的发生率由90%降至5.8%，在国外被广泛采用。并被《美国手汗症治疗专家共识》（2011年版）引用。福建医大一院迄今已完成电视胸腔镜胸交感神经切断术治疗手汗症2, 200余例，手术量居全国首位，有效率达到100%。不少国外的医生前来参观学习。

## 10 关于我国胸外科专业数据库的现状分析

由于缺少前瞻性数据库，缺少数据管理人才，医师缺乏建立、使用数据库的训练，术后长期随访成为我国临床医学各专业最困难的事。而建立专业数据库是临床科研的原始资料的积累、疾病诊治指南的依据和政府卫生决策的主要参考。目前国际主要医疗数据库有监测-流行病学及结论组织（Surveillance, Epidemiology, and End Results Program, SEER）检测流行病学及结论组织、国际肺癌研究协会（International Association for the Study of Lung Cancer, IASLC）、美国胸外科医师协会（The Society of Thoracic Surgeon, STS）、国际外科质量改进计划（National Surgical Quality Improvement Program, NSOIP）和国家癌症数据库（National Cancer Database, NCDB）。这些著名的医学数据库为国际医学研究做出了突出的贡献。我们以2014年初在美国召开的STS会议上的一篇论文：“手术在国家癌症数据库75, 000例临床Ⅲ期原发性肺癌患者中的作用”看医疗大数据的重要性。目前手术治疗临床Ⅲ期NSCLC存在争议。作者从国家癌症数据库（National Cancer Data Base, NCDB）中筛选出75, 353例临床Ⅲ期的NSCLC患者（T1-4N2M0），其中仅8%的患者接受手术治疗。手术患者5年生存率为28%、非手术患者5年生存率为8%、未治疗患者5年生存率为4%（*P*＜0.001）。而在未行术前治疗的3, 819例患者中有38%患者术后病理降为N0或N1，即这些患者术前的临床诊断高估了N分期。论文的结论是：临床分期常较病理分期高估了N2的转移情况，使部分Ⅲ期患者失去了手术机会。由于这项研究采用的是大数据资料，使研究结果具有非常高的可信度。目前我国胸外科专业数据库仅在一些大的医疗单位各自建立，水平也参差不齐。只有各大医院胸外科都建立了自己的数据库，并与全国性的数据库相连接，我们才能得到非常宝贵的大数据，从而为医疗指南的建立、修改提供有说服力的证据。这是一项亟待进行的非常艰巨、非常困难而又非常重要的工作。在我国一些大医院胸外科已经建立的数据库中，北京大学肿瘤医院胸外科（陈克能）自2000年1月开始建立的数据库是非常先进的一个。在该科数据库中，术前资料内容中包括一般信息、既往史、术前检查报告、新辅助治疗资料、影像学资料、病历等内容；手术资料内容中包括手术录像、标本、手术记录；术后资料内容中包括并发症记录、辅助治疗、随访资料等。随着新的病例资料的输入，并发症发生率、远期生存率等结果可随时得出最新数据。随访率达到了97.8%，这在我国临床专业中是非常难得可贵的，值得在全国推广。

